# Mitigating Barriers to Participant Recruitment and Retention in the READD‐ ADSP Programme: Experience from the African Dementia Consortium

**DOI:** 10.1002/alz.093147

**Published:** 2025-01-03

**Authors:** Rufus O. Akinyemi

**Affiliations:** ^1^ Neuroscience and Aging Research Unit, Institute of Advanced Medical Research and Training, College of Medicine, University of Ibadan, Ibadan Nigeria

## Abstract

Achieving greater diversity and inclusion in global dementia research requires the inclusion of underrepresented geographic, ethnic and regional populations such as indigenous Africans. The ADSP is a collaborative global initiative that includes sample collection across diverse populations, data generation including whole genome sequencing in over 120,000 individuals and multi‐omics, the collating of rich phenotypic information, data harmonization, and unified data management and quality control. These datasets are being analyzed to accelerate our understanding of AD neurobiology with implications for better risk prediction and discovery of novel precision diagnostics and therapeutics. The Recruitment and Retention for Alzheimer’s Disease Diversity Genetic Cohorts in the ADSP (READD‐ADSP) is a case/control study that is recruiting, retaining and evaluating 13,000 participants of diverse race/ethnicity, including 5,000 indigenous Africans (AF), 4,000 Black Americans (BA), and 4,000 US Hispanic/Latinx (HL). The study in Africa involves multiple sites in 9 African countries operating under the African Dementia Consortium (AfDC). Barriers and facilitators of recruitment have been observed. A total of 652 participants (302 AD cases and 352 controls) have been recruited into the READD – ADSP Africa programme between 22nd June, 2023 and January 22^nd^ January, 2024 from diverse populations in 8 countries in eastern, central and western Africa. Identified barriers to recruitment include non‐existence or poorly managed dementia registries, language diversity, myths around blood donation for research, family dynamics, stigma and unmet expectations regarding incentives for participating in research and concerns about data privacy. These challenges were mitigated through context ‐ sensitive community – engagement programmes involving community advisory boards, leveraging previous research programmes and existing doctor – patient relationships, and use of social media. The collaboration of the ADSP with the African Dementia Consortium (AfDC) to recruit participants into the READD – ADSP program is yielding positive results with progressive recruitment of study participants while innovative and context – sensitive stakeholder engagement strategies are mitigating the barriers to participant recruitment and retention.

